# Oxidic 2D Materials

**DOI:** 10.3390/ma14185213

**Published:** 2021-09-10

**Authors:** Oliver Dubnack, Frank A. Müller

**Affiliations:** 1 Otto Schott Institute of Materials Research (OSIM), Friedrich Schiller University Jena, Löbdergraben 32, 07743 Jena, Germany; oliver.dubnack@uni-jena.de; 2 Center for Energy and Environmental Chemistry Jena (CEEC Jena), Friedrich Schiller University Jena, Philosophenweg 7a, 07743 Jena, Germany

**Keywords:** ultra-thin films, two-dimensional, monolayer, transition metal oxides

## Abstract

The possibility of producing stable thin films, only a few atomic layers thick, from a variety of materials beyond graphene has led to two-dimensional (2D) materials being studied intensively in recent years. By reducing the layer thickness and approaching the crystallographic monolayer limit, a variety of unexpected and technologically relevant property phenomena were observed, which also depend on the subsequent arrangement and possible combination of individual layers to form heterostructures. These properties can be specifically used for the development of multifunctional devices, meeting the requirements of the advancing miniaturization of modern manufacturing technologies and the associated need to stabilize physical states even below critical layer thicknesses of conventional materials in the fields of electronics, magnetism and energy conversion. Differences in the structure of potential two-dimensional materials result in decisive influences on possible growth methods and possibilities for subsequent transfer of the thin films. In this review, we focus on recent advances in the rapidly growing field of two-dimensional materials, highlighting those with oxidic crystal structure like perovskites, garnets and spinels. In addition to a selection of well-established growth techniques and approaches for thin film transfer, we evaluate in detail their application potential as free-standing monolayers, bilayers and multilayers in a wide range of advanced technological applications. Finally, we provide suggestions for future developments of this promising research field in consideration of current challenges regarding scalability and structural stability of ultra-thin films.

## 1. Introduction

Crystalline thin-films of complex oxide materials possess a wide range of fascinating physical, electronic, chemical and optical 2D correlated properties which can be tuned due to their stoichiometry- or composition-dependency and deviate entirely from the behaviour of the bulk solid due to the reduction of dimensions and symmetry [1,2,3]. They include phase transitions and nanoscale elasticity [4], unconventional high-temperature superconductivity [5,6,7,8,9,10,11], colossal magnetoresistance [12,13], Mott metal-insulator transitions [14,15], multiferroicity [16,17,18] and other exotic magnetic properties [19] as well as strong light interaction [20] and distinctive electronic properties as a result of strong electron-electron correlations [21]. For example, as the layer thickness is reduced approaching the monolayer limit, the band structure and the mechanical properties such as flexibility and strength of the material change. Furthermore, thin films are often characterized by high electron mobility, high thermal conductivity and optical transparency due to strong in-plane covalent bonds and atomic layer thickness [22,23,24,25]. The discovery of unconventional 2D correlated quantum phases and flexoelectric/flexomagnetic effects [26] also contributes to the potential of ultra-thin oxide films for electronic applications [27,28,29,30] and the development of next-generation multifunctional devices in the fields of electronic, spintronic, magnetoelectric, neuromorphic and energy conversion storage [31,32,33,34].

Useful interface functions can be further boosted by stacking layers of unusual material combinations to create heterostructures that allow the targeted adjustment of electronic properties, strongly modify the optical properties [35] and evoke exotic quantum phenomena [20].

In 2017, Tan et al., hypothesized that, with the help of suitable experimental conditions, the production of any form of two-dimensional materials is possible, provided that their growth can be restricted to two dimensions and a few atomic layers [2]. However, it has been assumed that there is a critical limit to the minimum film thickness for stabilizing crystalline order in ultra-thin films, below which the membrane lattice of the film would collapse via chemical bond breaking, making it impossible for most materials to form stable monolayers [36]. Beyond that, crystalline oxides are composed of strong covalent and/or ionic 3D bonds. Consequently, there is no possibility to exfoliate individual sheets of layered compounds with weak out-of-plane bonds [36], as it is done for example in the production of graphene, the most famous 2D material [4]. The isotropic bonding between atoms or molecules not only complicates the exfoliation of three-dimensional oxide crystals, but also the detachment of strongly bonded ultra-thin oxide films from their substrate [26].

**Figure 1:**
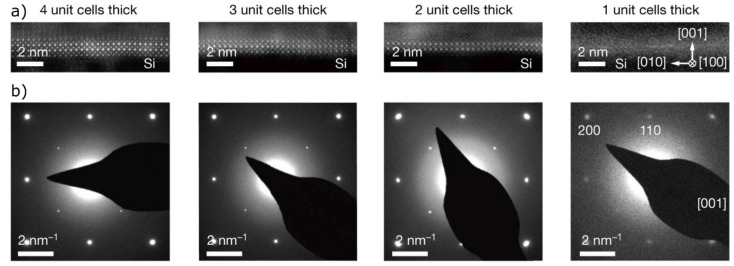
(**a**) Cross-sectional high-angle annular dark-field (HAADF) images and (**b**) selected area electron diffraction (SAED) patterns of ultra-thin, free-standing SrTiO_3_ films of different unit cell thicknesses. Reprinted from Ji et al. [26] by permission from Springer Nature. Copyright 2021.

However, scientists have recently succeeded in synthesizing crystalline quality perovskite films with a minimal thickness down to a single unit cell, which represents the smallest repeating unit of a periodic lattice, by using a bottom-up layer-by-layer technique (Figure 1a,b) [26,37,38]. Beyond that, they were able to exfoliate these monolayers by using intermediate layers of graphene [38] or a water-soluble oriented single crystal with similar lattice parameters as the target crystal [26,37] to produce free-standing single-crystalline films of complex oxide materials and to transfer them to other substrates. These findings greatly expand the technological potential of 2D oxides for the development of multifunctional electronic applications and at the same time raise a number of further questions regarding the transfer process and its extension to other materials and structures.

Here, we describe the state-of-the-art scientific knowledge and at the same time combine it with visionary thoughts regarding the issue of monolayers and ultra-thin films. This review describes the characteristics of promising oxidic 2D materials and strategies for their fabrication and transfer. Application fields for ultra-thin films are elaborated to illustrate their potential and future perspectives.

## 2. 2D Materials

2D materials are defined as sheet-like solid crystals composed of a single or few atomic layers [2]. They are typically free-standing [22,39,40] and have a thickness of 1–10 Å. Ramesh et al. define three types of thin-film architectures, including (i) single-phase epitaxial thin films, i.e., single-crystalline films having their growth orientation dictated by the orientation of a single-crystalline substrate material, (ii) horizontal heterostructures in which a magnetic phase is epitaxially interleaved with a ferroelectric (piezoelectric) phase and (iii) nanoscale ‘vertical heterostructures’ as the vertical analogue [16]. An additional subdivision into three classes, that can be prepared in the form of single-atom- or single-polyhedral-thick layers, is given by Butler et al. [22]: (i) layered van der Waals solids contain individual layers bonded together by van der Waals forces, in which the atoms are covalently or ionically bonded, (ii) layered ionic solids represent crystal structures consisting of charged 2D polyhedral layers held together by ionic bonding, and (iii) multilayer assemblies produced by electrostatic layer-by-layer deposition or self-assembly processes.

### 2.1. Non-Oxidic 2D Materials

The first breakthrough regarding two-dimensional materials was achieved by Novoselov and Geim in 2004 by the mechanical exfoliation of graphite to form individual graphene layers [41]. The crystalline monolayer of carbon atoms arranged in a two-dimensional honeycomb lattice exhibits numerous optical, mechanical, thermal and electrical property phenomena [23,27,42,43,44], offering a wide range of applications, including high-speed electronics [45], optical devices [46], energy generation and storage [46,47,48] and chemical sensors [23,49]. Of particular interest are the superconducting properties of graphene thanks to its electron mobility just below the theoretically predicted limit [42]. These can be further modified both by stacking two graphene layers that are twisted relative to each other by a ‘magic angle’ [50] and by intercalating different ions [51].

Driven by the great interest in thin films with exotic properties, further layered 2D inorganic materials have been the subject of scientific research in recent years. One of the most important classes of these materials are the transition metal dichalcogenides (TMD), which consist of hexagonal layers of metal atoms (M = Mo, W, Nb, Re, Ni or V) sandwiched between two layers of chalcogen atoms (X = S, Se or Te) with a MX_2_ stoichiometry [52]. The applications of TMD range from atomically thin semiconductors [53] and transistors [28] to ultra-sensitive photodetectors [29], optoelectronics and energy harvesting [28].

Other non-oxidic 2D representatives include hexagonal boron nitride insulators [54], two-dimensional metal carbides and nitrides (MXenes) [55] and topological insulators consisting of Bi_2_Te_3_, Sb_2_Se_3_ and Bi_2_Se_3_, respectively [56,57]. The structural and property diversity of these materials allows the exploration of new phenomena [58] that are not possible in graphene due to the lack of forbidden energy regions [59]. The common feature of all these materials is their layered structure with strong chemical bonds in two dimensions and significantly weaker van der Waals interactions along the perpendicular direction [22,60].

Numerous high-performance applications for ultra-thin non-oxidic materials are emerging due to their promising coexisting properties, ranging from electronics and optoelectronics [24,25,28,42,61,62] to energy storage [63,64,65] and sensors [66,67,68,69,70]. Nevertheless, the potential of 2D layers could be significantly extended by considering oxide materials and their structural characteristics.

### 2.2. Transition Metal Oxides

Transition metal oxides (TMO) represent an ideal platform for the investigation of interfacial processes and electron correlations due to the dominance of strongly correlated d electrons and the transfer of metal s electrons to the oxygen ions [71,72]. These d electrons exhibit more degrees of freedom [73] compared to conventional 2D materials and thus determine the physical properties of the given material. In addition, thin films of TMOs differ from the honeycomb lattice of graphene-like conventional 2D materials because they exhibit 3D lattices [74,75]. Highly tunable TMO interfaces are of great interest because of their diverse physical properties such as high temperature superconductivity, piezoelectricity, ferroelectricity, magnetoresistance and multiferroicity [76] which result from the correlation of atomic and structural degrees of freedom such as spin, charge, orbital and lattice [14,77]. Studies on the influence of film thickness and the effect of epitaxial strain on films [78,79] show, for example, a decrease of the electric polarization with decreasing film thickness [58] and a strong dependence of the piezoelectric stress and strain coefficients on an applied mechanical stress as well as on the material structure and composition [59].

As part of the TMO class of materials, so-called multiferroics represent 2D materials that are characterized by a combination of several primary ferroics, in particular ferromagnets (spontaneous magnetization that is switchable by an applied magnetic field), ferroelectrics (spontaneous electric polarization that is switchable by an applied electric field) and ferroelastics (spontaneous deformation that is switchable by an applied stress) [16,80,81]. Multiferroics, by restricting their geometry to two dimensions, open up promising possibilities to explore fascinating property phenomena.

#### 2.2.1. Perovskites

**Figure 2:**
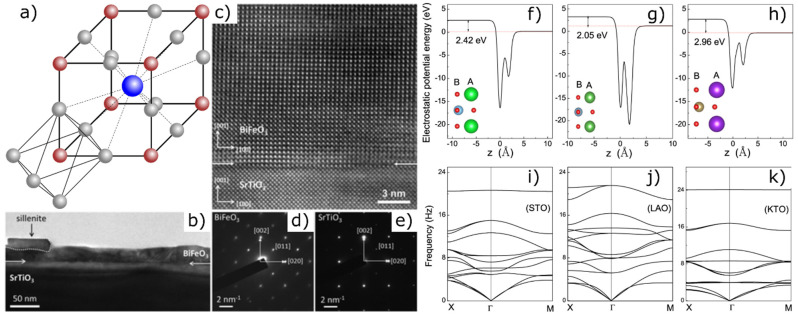
(**a**) Unit cell of perovskite ABO_3_ (A: blue, B: red, O: grey). (**b**) Low-magnification transmission electron microscopy (TEM) image of a BiFeO_3_ thin film grown on (001)-oriented SrTiO_3_. (**c**) Fourier-filtered TEM image of the interface between BiFeO_3_ and SrTiO_3_ showing no misfit dislocations. (**d**,**e**) SAED patterns of the BiFeO_3_ film. Reprinted with permission from Akbashev et al. [100]. Copyright 2021 American Chemical Society. (**f**–**h**) Intrinsic average electrostatic potentials with optimized atomic structure and (**i**–**k**) phonon dispersion spectra of the three monolayers SrTiO_3_ (STO), LaAlO_3_ (LAO) and KTaO_3_ (KTO), studied by the first-principles method [105]. © IOP Publishing. Reproduced with permission. All rights reserved.

Perovskites represent one of the most important crystal structures among TMOs. They are generally composed of six oxygen ions, which generate a crystal field that acts on the surrounded transition metal ion [71]. With the general formula ABO_3_, where the A site is occupied by the larger charge balancing metallic cation compared to the B site, the perfect perovskite structure results in a cubic symmetry lattice [82,83] with the B cation located in the centre of corner-sharing BO_6_ octahedral building blocks [2] (Figure 2a). Perovskite unit cells typically have an edge length of 0.4 nm [83]. Besides calcium (A cation) and titanium (B cation), which constitute the typical perovskite structure calcium titanate (CaTiO_3_), perovskites can also contain numerous other metals. Calcium, for example, is replaced by alkali metals, rare earth metals or iron, while titanium is often substituted by niobium and, to a lesser extent, by tantalum and zirconium.

In 1989, Matsubara et al., fabricated epitaxially grown films with a thickness of several hundred nanometers from AB0_3_ oxides such as PbTiO_3_, BaTiO_3_ and SrTiO_3_, by magnetron sputtering [84]. Furthermore, thin films of perovskite-type oxides were deposited by pulsed laser deposition (PLD) or grown by sputtering to analyze the temperature dependent colossal magnetoresistance [12,85], twinning effects [86] and the influence of deposition parameters and post-deposition heat treatments on relevant properties [87]. In 1998, Gu et al., succeeded in growing single-layer perovskite Bi_4_Ti_3_O_12_ thin films by using a sol-gel process [88]. In the following years, numerous studies dealt with the stability of ferroelectric states in perovskite films with thicknesses ranging from a few unit cells to several hundred Å [89,90,91,92]. SrTiO_3_ and BiFeO_3_ as typical representatives of the perovskite class of material were often used to analyze the preparation of thin films and their properties [37,93,94,95,96,97,98,99,100]. Akbashev et al., report an atomic layer deposition (ALD) process that enables the epitaxial stabilization of high-quality, phase-pure, single-crystalline, epitaxial and dislocation-free BiFeO_3_ (001) thin films on SrTiO_3_ (001) (Figure 2b–e) [100]. Furthermore, materials like PbTiO_3_ [101], (1-x)BaTiO_3_-xBi(Mg,Ti)O_3_ [102], La_1-x_Sr_x_CrO_3_ [103] and BaTiO_3_ [104] were the subject of extensive investigations on thin films.

Strontium titanate (SrTiO_3_), a ternary transition metal oxide, represents one of the most studied thin film materials. Exhibiting a simple cubic crystal structure (space group: Pm3m, a = 3.9 Å) with mixed ionic-covalent bonding character, the perovskite consists of alternating TiO_2_ and SrO planes [82,106,107]. The permittivity of this ‘superconducting semiconductor’ [108] with an indirect band gap of 3.25 eV [99,109] is tunable within wide limits via cationic doping [99,110,111,112,113], oxygen vacancies [114,115] and field effects [116,117], extending the achievable range of transport properties. Since epitaxial coherency strains have the ability to stabilize ferroelectricity in SrTiO_3_ thin films [112,118,119,120], an increase in the superconducting transition temperature can be observed compared to unstrained films grown under the same conditions [121]. The semiconductor properties of SrTiO_3_, its thermal and physiochemical stability and its non-toxicity make the material appealing for applications including photovoltaic cells, ferroelectrics and thermoelectrics [82,122,123,124,125,126,127], as well as for modern electronic and optoelectronic applications [105].

Bismuth ferrite (BiFeO_3_) represents an intensely investigated single-component multiferroic [16,78,95]. While bulk single crystals usually crystallize as rhombohedral distorted perovskites (space group: R3c, a = 3.96 Å) [78,95,98], the growth of thin films may induce the formation of lower symmetry structures due to strain imposed by the substrate [78,95,96,98,128,129,130,131,132,133,134,135,136,137,138,139,140,141] and thus offer great flexibility with regard to the structural parameters. Semiconducting BiFeO_3_ films show a significantly lower room-temperature band gap (2.6–3.0 eV) compared to the majority of ABO_3_ perovskites [142,143,144,145,146]. The wide range of functional properties gives BiFeO_3_ the potential for application in ferroelectric non-volatile memories [16] and a variety of electronic (capacitor, storage media, memristors) and optical (plasmon resonator, thin film modulator, photovoltaic) devices, as well as in the field of spintronics [78,147].

The original assumption that a perovskite membrane lattice collapses below a critical thickness of five unit cells [36] was disproved in 2019 when Ji et al., used molecular beam epitaxy (MBE) to produce free-standing SrTiO_3_ and BiFeO_3_ perovskite films with high crystalline quality and a thickness of one unit cell [26]. Based on these results, Xiao et al., investigated tetragonal oxidic perovskite monolayers as 2D materials using the first-principles method. Apart from SrTiO_3_, they found LaAlO_3_ and KTaO_3_ to be two other stable, free-standing 2D monolayer materials (Figure 2f–k) [105].

In general, bulk oxidic perovskite materials display multiple physical effects, including piezoelectricity, multiferroic behaviour, high permittivity and conductivity, optical transparency, photocatalytic properties and colossal magnetoresistance [71,82], potentially useful for emerging functional materials and devices in photovoltaics, sensor technology or energy harvesting systems [16,148,149,150]. The wide range of properties results from the high flexibility of the chemical composition due to incorporation of different cations [83]. Since crystalline orientation and perovskite properties are closely related [39], physical characteristics such as the band structure, electron and hole transport properties, photoluminescence and dielectric behaviour may be affected by the degrees of tilting of the octahedra [151,152], the influence of strain relaxation or the preparation of free-standing thin films [39]. For instance, orientation-dependent ferroelectric and magnetic anisotropy have been detected in bulk BiFeO_3_ [153] and SrRuO_3_ [154,155].

Although it has been assumed for a long time that a critical layer thickness exists below which spontaneous polarization is suppressed, it has been found that polarization persists along the (111) plane [98] in one unit cell perovskite films [156] and even in free-standing thin films [26]. In addition, the reduction of the thickness of thin epitaxial films increases the magnetization and the occurrence of a strong magneto-electric coupling [16]. Monolayers of the typical perovskites SrTiO_3_, LaAlO_3_ and KTaO_3_ represent 2D wide-gap semiconductors with indirect band gaps, where the band structure is very different from the corresponding bulk oxides. The large electrostatic potential energy difference that occurs between the top and bottom of the thin films induces a large out-of-plane dipole, leading to gating effects and to special optical properties of the materials [105].

#### 2.2.2. Garnets

**Figure 3:**
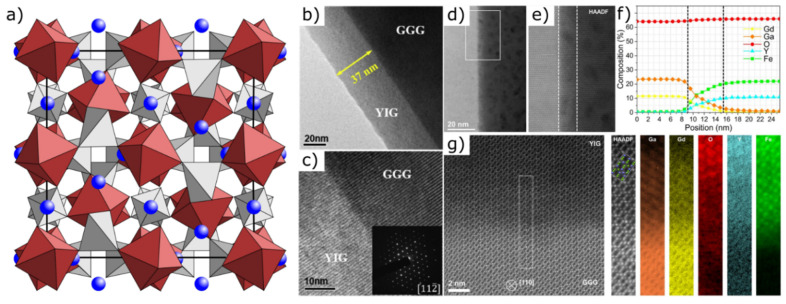
(**a**) Unit cell of garnet A_3_B_2_C_3_O_12_ (A: blue, BO_6_: red, CO_4_: grey). (**b**,**c**) TEM cross-section images of a YIG/GGG (111) thin film grown at 850 °C for 3 h with a thickness of 37 nm (inset: diffraction pattern of the interface). Reprinted from Ma et al. [168]. Copyright 2021, with permission from Elsevier. (**d**) HAADF overview image of a YIG/GGG interface (YIG: right, GGG: left). The white rectangle indicates the region over which the electron energy loss spectroscopy (EELS) imaging in (**f**) was carried out, providing averaged compositional profiles. (**e**) HAADF intensity of the YIG/GGG interface. White dotted lines indicate a ∼6 nm wide region where some interdiffusion of Gd, Y, Ga, and Fe is observed (corresponding to the area marked with dotted lines in (**f**)). (**g**) High magnification EELS analysis of the interface (rotated 90 degrees to (**d**)) in the region indicated by a white rectangle on the HAADF overview image. Maps for Ga, Gd, O, Y and Fe are shown, together with the simultaneously recorded HAADF intensity, over which a sphere model of YIG in [110] orientation is superimposed (green spheres represent Fe, blue spheres Y and oxygen is not shown for clarity) [163].

The structure of garnets shows a face-centred cubic lattice (space group: Ia3d, point group: n13m), whose unit cell consists of eight formula units A_3_B_2_C_3_O_12_ (A = Ca, Mg, Fe, Mn, etc., B = Al, Fe, Cr, V, etc., C = Si, As, V, Fe, Al, etc.) [157] (Figure 3a). Common species are pyralspite garnets (almandine Fe_3_Al_2_(SiO_4_)_3_, pyrope Mg_3_Al_2_(SiO_4_)_3_, spessartine Mn_3_Al_2_(SiO_4_)_3_) with aluminium on the B site as well as the ugrandite group (andradite Ca_3_Fe_2_(SiO_4_)_3_, grossular Ca_3_Al_2_(SiO_4_)_3_, uvarovite Ca_3_Cr_2_(SiO_4_)_3_) with calcium on the A site. Depending on the composition, garnets usually have ferrimagnetic properties and a comparatively high hardness.

The decisive criterion for the production of thin films is the dependence of all characteristic properties of crystals with garnet structure on the lattice parameter. If this parameter increases, there is an increase in elastic interactions, accompanied by a weakening of interatomic bonds [157]. As early as 1958, Dillon et al., reported on 25 µm thick garnet films for the investigation of magnetostatic modes and power saturation effects. They used the transmission properties of the magnetic material to distinguish domains of different magnetization [158]. In 1967, Mee et al., documented the growth of single-crystalline epitaxial yttrium iron garnet (YIG) and gadolinium iron garnet (GdIG) films with a thickness of 2–3 µm using chemical vapor deposition (CVD) [159]. Krumme et al., investigated thermomagnetic flux reversal in 5 µm thick single-crystal layers of Y_3_Gal_1.1_Fe_3.9_O_12_, prepared by liquid-phase epitaxy [160]. In the following years, both optical [161] and magnetic [162] properties of garnet films of different composition were analyzed. Structural and magnetic characterizations of single crystal thin-film YIG revealed a 4–6 nm thick interdiffusion zone at the YIG-substrate (gadolinium gallium garnet, GGG) interface (Figure 3d–g) [163]. The occurrence of magneto-optical effects affecting the Curie temperature, growth-induced uniaxial anisotropy, optical absorption behaviour and refractive index confirmed the interest in using thin films with garnet structure in applications such as displays, printers and components for optical communication [164,165,166,167].

In 1997, Levy et al., succeeded in epitaxial lift-off of magnetic Y_3_Fe_5_O_12_ (YIG) grown by liquid-phase epitaxy. They produced a buried sacrificial layer by deep-ion implantation for the lift-off of 10 µm-thick films of excellent single-crystal quality and bonded them to various substrates [169]. Although YIG films are normally quite brittle, crack free materials could be obtained after the lift-off. Neither ion implantation nor the lift-off procedure caused changes in the shape or size of the domains. Using pulsed laser deposition, Popova et al., were able to produce ultra-thin polycrystalline YIG films (100–500 Å) at substrate temperatures above 400 °C to reproduce the structure of the target and avoid the formation of amorphous membranes [170]. With decreasing film thickness, an increasing deviation of the magnetic and opto-magnetic behaviour from the properties of the bulk material has been observed. For example, decreasing film thickness causes a significant decrease of the saturation magnetization. Furthermore, variations in the oxygen partial pressure lead to changes in lattice deformation, surface roughness and film thickness.

Epitaxial single crystalline YIG thin films can be deposited on (111)-oriented GGG (Figure 3b,c) or other garnet substrates at different temperatures via sputtering processes [168]. The stabilization of the garnet phase and an increase in transmittance via doping to improve the material’s magneto-optical performance [171] as well as possibilities to control perpendicular magnetic anisotropy [172] and magnetization dynamics by substrate orientation [173] have recently been demonstrated. Kotov et al., achieved an important step towards the fabrication of high-performance ultra-thin garnet films by using magnetron sputtering deposition and crystallization annealing for the growth of magneto-optical bismuth-substituted iron-garnet films [174]. The crystallization behaviour of the garnet layer was supported by the deposition of a thin protective bismuth oxide (Bi_2_O_3_) layer, which led to a strong increase in magneto-optical quality with record-low optical losses in the ultra-high frequency spectral region, confirming the material’s potential for the development of spintronics and modern microwave devices. Such magneto-optical garnets could play an important role in the future development of ultra-fast optoelectronic devices, spintronics and modern microwave devices [175]. Various nano-electronic device types [176] and applications in photonics [177,178] are conceivable.

#### 2.2.3. Spinels

**Figure 4:**
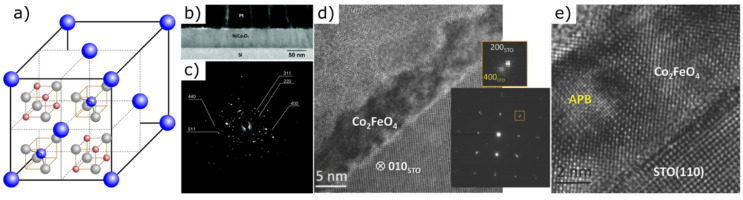
(**a**) Unit cell of spinel AB_2_O_4_ (A: blue, B: red, O: grey; orange cubes are also included in the rear half of the unit cell). (**b**) Low-magnification TEM image and (**c**) SAED pattern of a (Co_1-x_Ni_x_)_3_O_4_ film (x = 0.33). Republished from Hagen et al. [184] with permission of Royal Society of Chemistry. Copyright 2021. Permission conveyed through Copyright Clearance Center, Inc. (**d**,**e**) Cross-sectional TEM images of Co_2_FeO_4_ samples grown on (**d**) SrTiO_3_ (001) (inset: corresponding SAED pattern) and (**e**) SrTiO_3_ (110), viewed along the zone axis [010] of SrTiO_3_ [189].

Spinels are cubic crystals with the general structural formula AB_2_O_4_. Their large unit cell contains 8 A (= Mg, Zn, Fe, Mn, Mg, Cu, Ni, Ti, etc.), 16 B and 32 oxygen atoms (Figure 4a). On the basis of the B cation, spinels are grouped together, including in particular aluminum, iron, chromium, cobalt and vanadium spinels. The variety of remarkable physical, chemical, electrical and magnetic properties of spinels can be explained by the distribution possibilities of the cations in spinel bulk crystals [179]. This results in superior chemical stability and mechanical hardness [180] in certain cases. Magnetic anisotropy can be incorporated by Co ions [181]. Lattice dimensions of spinels correlate directly with the composition [179].

Miikkulainen et al., reported on the production of uniform polycrystalline lithium manganese oxide spinels by atomic layer deposition. Due to its high electrochemical capacities, structural cycling stability, low costs and high safety, this material appears promising for its use in thin-film lithium-ion batteries [182]. Other representatives of spinel oxides, which were also produced as high-quality crystalline thin films using the ALD process, include Li_4_Ti_5_O_12_ [183] and (Co_1-x_Ni_x_)_3_O_4_ (Figure 4b,c) [184]. The latter was also grown using PLD [185] and has potential applications in photovoltaics, spintronics and thermoelectrics. Suzuki et al., grew high-quality crystalline CoFe_2_O_4_ and (Mn,Zn)Fe_2_O_4_ spinel films with a thickness ranging from 700 to 3000 Å using the PLD method [186]. Only by using spinel structured buffer materials, namely CoCr_2_O_4_, CuMn_2_O_4_, FeGa_2_O_4_ and NiMn_2_O_4_, highly crystalline ferrite films with bulk magnetization properties could be produced. Uhrecky et al., successfully achieved the growth of Ba_2_Zn_2_Fe_12_O_22_(Y) ferrite using a chemical solution deposition method [187], while Lüders et al., reported on the epitaxial growth of spinel NiFe_2_O_4_ ultra-thin films [188] showing an enhanced magnetic moment and a metallic character in comparison to the bulk material. In 2014, Coll et al., produced ultra-smooth and pure magnetic Co_2_FeO_4_ thin films with a thickness of 5–25 nm using ALD (Figure 4d,e) [189]. The increase in magnetization and coercivity compared to the bulk crystal of the Co-rich spinel ferrite is maintained to a film thickness of 10 nm and is lost when the thickness is further reduced due to the high density of structural defects. Heteroepitaxial stabilization leads to the formation of fully relaxed films showing high coercive fields and a high saturation magnetization. It is suggested that inducing epitaxial stabilization by the use of a substrate with smaller lattice mismatch would further improve the magnetic properties. Instead of traditional thin-film deposition techniques that require high processing temperatures or post annealing treatments to achieve such characteristics, this low-temperature and low-cost epitaxial growth process offers promising opportunities regarding future applications of Co_2_FeO_4_ films which include sensors, microelectronics and spintronics.

In general, spinel-type ferrite oxide materials are very flexible for application as electromagnetic devices due to their remarkable electrical and magnetic properties [180] and are used as conductors, dielectrics, resistors or magnetic sensors [190,191,192]. Compared to garnets, ferrite films are characterized by low conductivity and high saturation magnetizations and Curie temperatures, which makes them ideal for the use in high frequency applications [186].

However, limiting factors for the use of 2D materials, apart from their quality and quantity, are production yield and poor long-term stability. In order to meet commercial requirements, the problem of mass production of ultra-thin films must therefore be solved in the future.

## 3. Fabrication Strategies

### 3.1. Growth Techniques

Methods for synthesizing thin films can be divided into two categories, top-down and bottom-up approaches. The former are based on exfoliation of individual layers from a layered bulk crystal and are probably the simplest non-destructive technique for the fabrication of ultra-thin 2D materials. This form of micromechanical cleavage using Scotch tape ensures perfect crystal quality and also has other advantages such as wide applicability and large lateral size of exfoliated layers [27,193,194,195]. However, it is a process with a low production rate and a relatively low yield. At the same time, this manual production method offers only limited possibilities to adjust the size, thickness and shape of the thin films with sufficient precision and repeatability. Top-down approaches can be used for graphene and other conventional 2D materials, but not for complex oxides because they do not have a layered structure.

Bottom-up approaches are based on the chemical reaction of individual precursors, self-assembly or epitaxial growth [83], where thin films are deposited on the surface of crystalline substrates acting as a starting material [4]. In addition to these epitaxial growth targets, the formation of thin layers is characterized by a weak van der Waals bond to the substrate, referred to as van der Waals epitaxy [22]. In general, epitaxial films can be divided into homoepitaxial (grown on a substrate of the same material) and heteroepitaxial (grown on a substrate of a different material) layers [196]. Freund et al., define three typical modes of film growth: (i) two-dimensional layer-by-layer growth with thin films growing layer by layer (the Frank-van der Merwe mode), (ii) three-dimensional island growth with individual islands growing directly on the substrate (the Volmer-Weber mode) and (iii) the 2D-3D island-on-layer growth representing a combination of the previous two growth modes, with the growth of three-dimensional islands taking place on a previously formed thin wetting layer (the Stranski-Krastanov mode) [197]. From this variety of nucleation and growth models [198], the layer-by-layer growth will be highlighted as one of the most common modes. In a first step, impinging atoms move on the substrate surface until they are immobilized at an energetically favorable position [198] and nucleate into 2D islands that act as steps to which further arriving atoms can attach to complete the monolayer and create a smooth surface [196]. Optimization of the deposition conditions by e.g., the substrate temperature and ambient gas pressure must also be considered as well to obtain the desired two-dimensional growth [199]. However, the requirement that the nucleation of each subsequent layer occurs only after the previous layer is completed is never achieved [198,200]. Under constant conditions, the nucleation of new islands on the underlying incomplete layer cannot be avoided once a critical island size is exceeded [201]. Near-perfect layer-by-layer growth and accompanying atomically sharp interfaces can be achieved by delaying the nucleation of new islands until the growing underlying monolayer is completed [198]. Rijnders et al., developed a kinetic growth manipulation method that involves rapid deposition of the required amount of material to complete one monolayer. In the following interval, the deposition is interrupted to allow reorganization of the deposited film [200].

#### 3.1.1. Pulsed Laser Deposition

**Figure 5:**
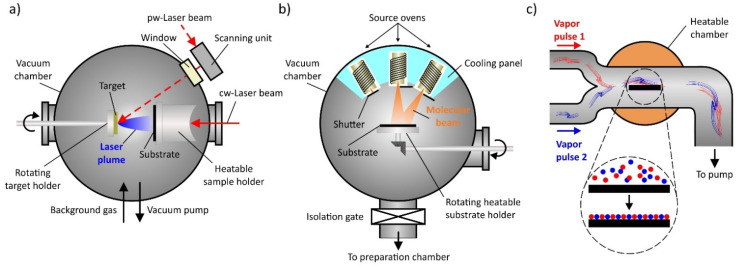
Schematic illustration of techniques for the deposition of ultra-thin films. (**a**) Pulsed laser deposition (PLD), (**b**) molecular beam epitaxy (MBE) and (**c**) atomic layer deposition (ALD).

Pulsed laser deposition (PLD) represents a layer-by-layer thin film fabrication technique to prepare complex oxide heterostructures (combination of individual thin films to create defined interfaces), superlattices (periodically layered structure of at least two materials) and controlled interfaces [198,202]. The PLD process is characterized by the use of a laser radiation source, typically a high-energy KrF excimer laser with a wavelength of 248 nm and pulse durations in the nanoseconds range [108,198]. The pulsed laser radiation is focused on a rotating target, ablating the target material by local heating and forming a high-energy expanding plasma [198,203]. The plasma and the atoms and ions it contains from the target spread out in vacuum and condense on the surface of a (heated) substrate, which serves as a nucleation site for the epitaxial growth (Figure 5a).

A large number of studies address the influence of controllable PLD growth parameters on the formation of thin oxide layers [76,99,107,198,203,204,205,206]. Important parameters affecting the PLD process are the laser power, background pressure and the distance between the target and the substrate. Shape and size of the plasma plume are influenced by the background pressure and therefore affect the deposition rate and film homogeneity [206]. The background gas can also be used to incorporate oxygen into the film or to improve its quality by preventing bombardment of the thin film by high-energetic plasma particles [206] and delaying scattering events on the trajectory [107]. Increasing the substrate temperature improves surface diffusion and growth kinetics of impinging species, resulting in enhanced 2D growth and suppressed incorporation of defects [107].

A major advantage of PLD compared to most other deposition techniques is the ability to set a desired stoichiometry of the thin film due to stoichiometric material removal at the target [198]. Nevertheless, deviations between the stoichiometric ratios of target and thin film, e.g., due to preferential scattering of lighter ablated species [76], have to be considered. Furthermore, the stoichiometry shows a strong dependence on the laser fluence, which consequently also affects the structure and properties of the thin films [94,99,207]. PLD is a suitable method to synthesize complex oxide materials, obtain interfaces or spatial variations of the composition on a larger substrate, control lateral thickness variation or approximate temperature-gradients [198]. By taking advantage of the fact that a single laser pulse leads to the deposition of significantly less than a monolayer of material, it is also possible to alternate between different materials during the process to build heterostructures or enable superlattice growth [198,203].

#### 3.1.2. Molecular Beam Epitaxy

Molecular beam epitaxy (MBE) is a technique for growing high-quality epitaxial thin films based on a variety of materials, including oxides, but also semiconductors and metals [196]. The process is mainly used in semiconductor technology and benefits from the precise control of composition during growth. After thermal evaporation of various elements, the beam of atoms or molecules in an ultra-high vacuum environment is directed onto a heated crystal with an almost atomically clean surface, forming a crystalline layer. Its crystal structure strongly depends on the structural properties of the substrate, which provides sufficient thermal energy at elevated temperatures to ensure surface diffusion of the incoming atoms (Figure 5b).

Possible contaminants surrounding the growing crystal have a decisive influence on electrical properties, film morphology and growth behaviour. Therefore, the vacuum must be kept as high as possible [196]. The temperature of the sources also has to be controlled precisely to adjust the flux of all kinds of materials being involved in the growth process to obtain the desired film ratio [196]. Another important parameter is the substrate temperature, which affects the reaction rate of the species, their kinetics and both the composition and quality of the resulting film. During growth, the correct temperature window must be set to avoid amorphous or polycrystalline films due to insufficient diffusion energy (temperature too low) or the growth of 3D islands (temperature too high) [208].

MBE enables outstanding precision in adjusting the chemical composition of the growing films [209]. The ability to rapidly change the composition while maintaining stoichiometric ratios allows the fabrication of crystalline interfaces with almost atomic accuracy. Another advantage of MBE is the minimal contamination of the growing surface due to the cleanliness of the growth environment. This ensures the formation of structures that closely resemble idealized models of solid state theory [196]. Based on their studies on thin perovskite films, Brooks et al., found that the MBE method is able to produce slight off-stoichiometry films with lattice constants much closer to the bulk material compared to higher energy growth methods such as PLD [209].

Yang et al., successfully fabricated more than 10 kinds of perovskite oxide thin films and their heterostructures, including SrTiO_3_, BaTiO_3_, LaAlO_3_, LaTiO_3_ and others, using laser MBE, a technique combining the advantages of conventional MBE and PLD. Topography and lattice structure of the epitaxial films show atomic precision [210].

#### 3.1.3. Atomic Layer Deposition

One possibility to control the film thickness at the atomic level is offered by the technique of atomic layer deposition (ALD) as a special modification of chemical vapor deposition processes. The material to be deposited is chemically bonded to precursors and deposited as a thin film on the substrate surface in a self-limiting layer-by-layer growth mode [211,212]. The gaseous, liquid or solid precursors must be volatile and thermally stable to provide a sufficiently high deposition rate by reacting rapidly with surface groups or chemisorb on the surface [213]. In this stepwise repetition of self-limiting surface reactions, the layer thickness increases constantly in each deposition cycle (Figure 5c).

Compared to other techniques, ALD is characterized by the ability to deposit only 0.1 to 3 Å of material per cycle. The fact that only a fraction of a monolayer is deposited in one cycle means that, on the one hand, the process is very slow, but on the other hand, layer thicknesses can be set with atomic precision on large areas [211,212,213,214,215]. Apart from the accurate thickness control, ALD differs from other deposition methods in 100% conformality [213], low temperature and low vacuum deposition conditions [216,217,218]. Even on complex structures, excellent coverage can be achieved by ALD thin film coatings. Furthermore, thanks to atomic precision, optical, chemical and electronic properties of growing thin films can be precisely adjusted [219] and multilayer structures can be fabricated [213].

Drawbacks of this method include the risk of precursor contamination and low cost efficiency in the deposition of many technologically relevant materials, including several multicomponent oxides [211]. Furthermore, using the ALD method under low process temperatures for the production of epitaxial multicomponent oxide thin films inevitably leads to the need for a further annealing treatment [100,220,221].

### 3.2. Transfer

Since the thin films produced, regardless of the growth process, are initially bound to a defined substrate that served as an epitaxial growth target, their further use is severely limited. For the complete characterization of structure and properties as well as for the exploitation of the full application potential, the transfer of thin films to any substrate surface is inevitably necessary after completion of the growth process.

The major disadvantage of mechanical processes for the detachment of thin layers in the production of free-standing films is the unavoidable structural damage that is induced during this process. To achieve the goal of creating free-standing thin films, epitaxial growth of various single-crystalline materials is also possible on substrates that have undergone 2D modification, e.g., in the form of an additional coating. This allows a subsequent transfer of the thin film, but still ensures epitaxial growth guidance during the fabrication due to atomic potential fields of the substrate material, penetrating through the coating. In principle, the interaction of substrate and thin film is partially shielded by polar 2D materials, while the atomic interaction has a longer range for strongly polarized bulk materials. Consequently, the strength of the interaction can be selectively controlled by adjusting the polarity of the substrate and the interlayer [222]. Key advantages of these methods are the reusability of the substrate [37,223], the ability to fabricate free-standing oxide membranes of different orientations with minimal damage while retaining structural properties [37] and the possibility to realize flexible oxide-based electronic applications [104,224]. The great importance of free-standing thin films becomes clear when considering the limitations of heteroepitaxial growth methods, where growth can only be realized with approximately the same lattice constant or crystal structure and is thus severely limited [38].

**Figure 6:**
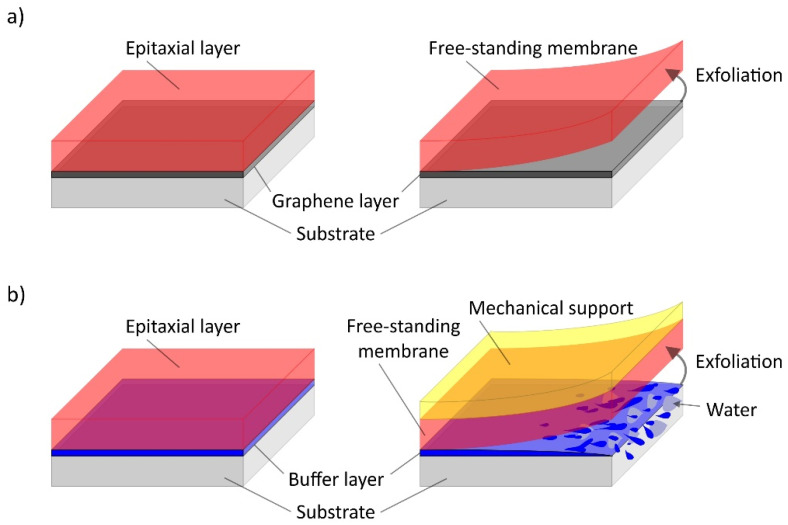
Approaches for transferring high-quality thin films. (**a**) Film and underlying substrate are separated by some weakly bonded graphene layers that transfer structural information. (**b**) Exfoliation after dissolution of an underlying water-soluble buffer layer and subsequent transfer using mechanical support.

A universal method for the generation of free-standing single-crystalline thin films of complex oxide materials is based on the separation of membrane and substrate by a few layers of graphene [18] (Figure 6a). The potential fields of the substrate atoms transmit crystal structure information through the graphene layer, thus serving as a growth guide for the growing thin film, which can subsequently be easily detached and transferred to any other material due to the weak van der Waals bonding forces between graphene and substrate. Calculations using density functional theory, according to which bilayer graphene has an optimal effect on achieving high crystalline quality, were confirmed using PLD grown SrTiO_3_ thin films on a graphene coated SrTiO_3_ substrate [38].

Epitaxial growth of water-soluble buffer layers using MBE [26] or PLD [39] represents another promising substrate pre-treatment for the fabrication of free-standing oxide membranes (Figure 6b). Subsequently, thin layers of the material to be deposited can be grown on the buffer layer via MBE or PLD. To allow subsequent removal of the thin film, the buffer layer is immersed in deionized water and dissolved [36,37,39] so that the thin film can finally be transferred to any other substrate using mechanical support [26,39]. Here, the selection of buffer layer and etchant is constrained by a variety of parameters, in particular etchant selectivity, correspondence of lattice symmetry for epitaxial growth and stability of the buffer layer [37].

Some representatives of oxide thin films could already be prepared by this method using Sr_3_Al_2_O_6_ buffer layers, e.g., BaTiO_3_ [224], YBa_2_Cu_3_O_7-x_ [223] and La_0.7_Sr_0.3_MnO_3_ [37]. Here, the aim should be to develop universal combinations of buffer layer and etchant, which can be generally used for the preparation of crystalline oxidic 2D membranes as well as their heterostructures.

## 4. Applications

Depending on whether 2D films are used as free-standing monolayers or stacked into bilayers or multilayers, a wide variety of property phenomena arise, which can deviate significantly from the behaviour of the bulk crystal, leading to a variety of different applications.

### 4.1. Free-Standing Monolayers

Free-standing films are particularly suitable to investigate surface and interface related material properties such as phase transitions and switchable polarization [26], unpredictable electronic properties [2] or nanoscale elastic behaviour [4]. The enormous flexibility resulting from a film thickness of a few unit cells opens the potential to develop flexible multifunctional electronic applications from these thin films [26].

**Figure 7:**
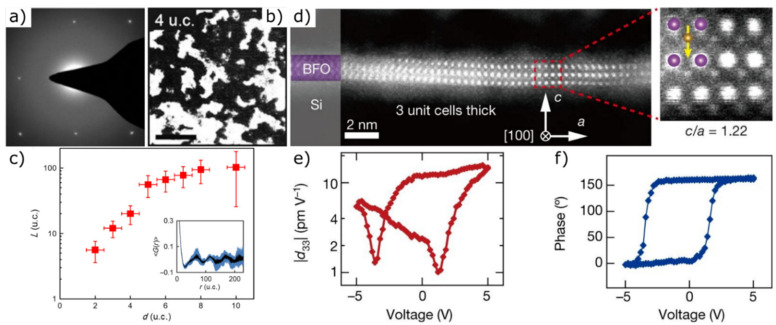
(**a**) Diffraction pattern and (**b**) dark-field TEM images (crystalline domains are shown in white) of 4-unit cell thick SrTiO_3_ membranes. (**c**) Thickness-dependent crystalline coherence length. Inset: Raw data of the spatial correlation function, derived from TEM images, from a 6-unit cell thick membrane [36]. (**d**) Cross-sectional HAADF images of a free-standing BiFeO_3_ film. (**e**) Piezoresponse force microscopy amplitude-voltage butterfly loop (d_33_: out-of-plane piezoelectric coefficient) and (**f**) phase-voltage hysteresis loop of a free-standing four-unit-cell BiFeO_3_ film on a conductive silicon substrate, showing that the polarization is switchable. Reprinted from Ji et al. [26] by permission from Springer Nature. Copyright 2021.

In 2017, Hong et al., succeeded in synthesizing single-crystalline SrTiO_3_ membranes using a SrTiO_3_ substrate and a Sr_3_Al_2_O_6_ buffer layer. Below a critical threshold of layer thickness of 4 unit cells, they observed the formation of a mixture of crystalline and amorphous regions (Figure 7a,b). Further reduction of the layer thickness to two unit cells led to the formation of an almost completely amorphous layer. The crystalline coherence length shows a continuous decrease with a qualitative trend change at a thickness below five unit cells (Figure 7c). According to their theory, the crystalline structure of SrTiO_3_ thin films is maintained as long as there is an epitaxial connection with a bulk substrate. The dissolution of the buffer layer and the resulting lift-off cause bond breaks at the interface, leading to the release of a large amount of free energy, which in turn triggers the crystalline-amorphous phase transition [36].

Contrary to this original assumption, Ji et al., 2019 succeeded in fabricating free-standing ultra-thin crystalline SrTiO_3_ and BiFeO_3_ films (Figure 7d) of high quality down to a layer thickness of a single unit cell by using MBE. Despite the strong ionic and covalent bonding forces that prevail in a three-dimensional oxide crystal, proof was provided for the first time that there is no critical limit to the minimum film thickness required to stabilize ultra-thin crystalline oxide films. Approaching the 2D limit, BiFeO_3_ exhibits a rhombohedral-tetragonal phase transition, large c/a ratios, enormous polarization and clear hysteresis loops, indicating that the polarization is switchable (Figure 7e,f). SrTiO_3_ films with a thickness of one or a few unit cells allow their use as a flexible ultra-thin oxide in multifunctional electronics due to their nanoscale elasticity behaviour [26].

### 4.2. Bilayers

Research into new physical and chemical properties as well as the development of functional applications is also possible by combining two layers to form heterostructured bilayers. The large amount of newly accessible properties creates entirely new application potentials and motivated initial investigations of thin film phenomena and considerations of their practical implementation [3,225,226].

Heterostructures of epitaxial, complex oxide thin films consist of a combination of membranes with different crystal structures and orientations, resulting in synergetic effects and a hybridization of physical properties. For example, the occurrence of charge redistribution and induced structural changes between neighbouring crystals could be observed [38]. Thus, the coupling of physical functionalities by stacking individual oxide layers enables novel applications. For example, bilayers are suitable for the production of ferroelectrics [227], spintronics [31,33] and energy conversion storage devices [32,34], supercapacitors [22], semiconductor device architectures [228] or flexible electronics [229] as well as multiferroics, piezoelectrics, magnetoresistors and superconductors [230]. The production of superconductive electronic devices such as transistors or microwave devices also seems promising [231]. However, to ensure widespread technological deployment, challenges such as surface reconstruction, charge transfers and built-in electric fields must be addressed [232].

**Figure 8:**
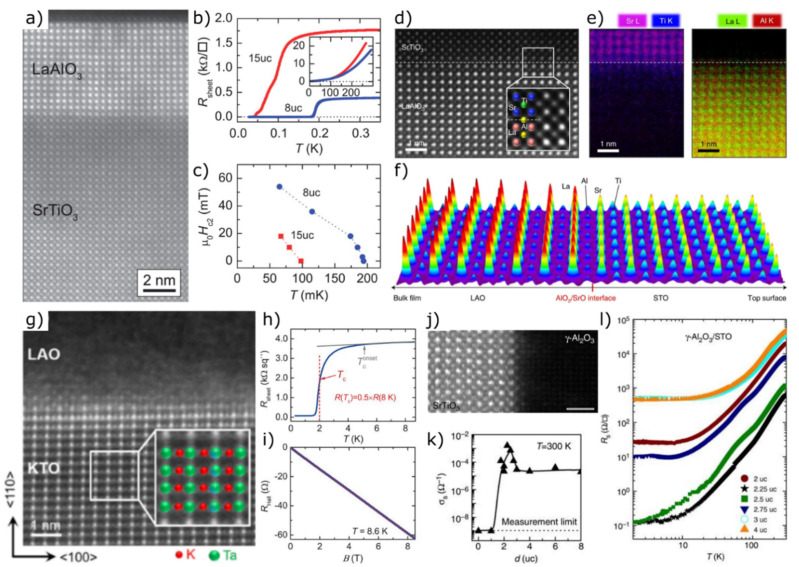
(**a**) HAADF image of the coherent interface between a 15-unit-cell-thick LaAlO_3_ film grown on SrTiO_3_. (**b**,**c**) Transport properties of LaAlO_3_/SrTiO_3_ bilayers with 8-unit-cell and 15-unit-cell-thick LaAlO_3_ films showing (**b**) the dependence of sheet resistance on temperature (inset: sheet resistance vs. temperature measured between 4 K and 300 K) and (**c**) temperature dependence of the upper critical field H_c2_. From Reyren et al. [9]. Reprinted with permission from AAAS. (**d**–**f**) Analysis of an epitaxially grown LaAlO_3_/SrTiO_3_ interface (**d**) STEM-ADF image (inset: filtered image with higher magnification and coloured atomic configuration). (**e**) Energy dispersive X-ray spectroscopy (EDS) elemental mapping showing an atomically abrupt interface without significant intermixing. (**f**) Coherent Bragg rod analysis (COBRA)-derived cation electron-density map showing an atomically abrupt interface consisting of SrO and AlO_2_ layers. Reprinted from Lee et al. [241] by permission from Springer Nature. Copyright 2021. (**g**) HAADF-STEM image of a 20 nm LaAlO_3_/KTaO_3_ (110) bilayer showing that the LaAlO_3_ film is amorphous (inset: image with higher magnification and coloured atomic configuration of KTaO_3_). Reprinted Figure 1a with permission from Chen et al. [253]. Copyright 2021 by the American Physical Society. (**h**) Dependence of the sheet resistance R_sheet_ on temperature and (**i**) dependence of the Hall resistance R_Hall_ on the magnetic field for a 20 nm LaAlO_3_/KTaO_3_ (111) device. From Chen et al. [257]. Reprinted with permission from AAAS. (**j**–**l**) Structural features and thickness-dependent electronic properties of an epitaxial spinel/perovskite γ-Al_2_O_3_/SrTiO_3_ interface. (**j**) HAADF-STEM image (scale bar: 1 nm). Sr ions are brightest, followed by Ti. (**k**) Thickness dependence of the sheet conductance σ_S_, measured at 300 K. A high-mobility 2DEG is obtained at a thickness between 2 and 3 unit cells. (**l**) Temperature dependence of the sheet resistance R_S_ at different film thicknesses. Reprinted from Chen et al. [141] by permission from Springer Nature. Copyright 2021.

The most prominent example in the field of bilayered oxide heterostructures is the combination of LaAlO_3_ and SrTiO_3_. Detailed reviews of experimental and theoretical work on growth conditions, dependence of electronic properties and structural features in LaAlO_3_/SrTiO_3_ systems are provided by Pauli et al. [233], Huijben et al. [234], Pentcheva & Pickett [235], Chen et al. [236], Zubko et al. [237] and Pentcheva et al. [238]. LaAlO_3_ films grown on SrTiO_3_ show a sharp and coherent interface without defects and dislocations (Figure 8a) [9]. Although both materials are wide bandgap insulators, a high-mobility two-dimensional electron gas (2DEG) forms at the interface of the LaAlO_3_/SrTiO_3_ thin films [9,71,239]. Two different models exist to describe the origin of the 2DEG, whereby only their combination allows a full explanation of all emerging phenomena. While the ionic defect mechanism is able to describe large parts of the observed physical properties [234], according to the polar mechanism a polar discontinuity between nonpolar SrTiO_3_ and polar LaAlO_3_ planes is the reason for the occurrence of 2DEG [240].

In 2018, a significant confirmation of the polar mechanism was provided when Lee et al., demonstrated the formation of a highly mobile two-dimensional hole gas (2DHG) that coexists with a 2DEG in epitaxial SrTiO_3_/LaAlO_3_/SrTiO_3_ heterostructures, grown by PLD [241]. The bilayer structure fulfils the requirements that are crucial for the interfacial charge confinement by avoiding atomic intermixing (Figure 8d). The existence of an atomically abrupt interface with atomic intermixing confined to about one unit cell was confirmed by energy dispersive X-ray spectroscopy (EDS) elemental mapping (Figure 8e) and coherent Bragg rod analysis (COBRA), indicating a sharp change in electron density at the interface (Figure 8f). The coexistence of 2DEG and 2DHG allows a more detailed investigation of confined electron-hole systems [241,242] and enables the development of new mesoscopic superconducting circuits [243]. The conductivity within the interfacial layer exhibits a strong dependence on the film thickness (Figure 8b,c) and on the surface termination of the individual SrTiO_3_ layers. The heterostructure is conductive for a TiO_2_ termination and insulating for a SrO termination [108,244] when a critical layer thickness of four unit cells is considered for the sandwiched crystalline LaAlO_3_ to enable conductivity [108,240,245]. The superconductivity can also be controlled by applying a gate voltage [243,246,247]. Beyond its exceptionally high conductivity, emergent magnetism was observed, although none of the materials is magnetic [108].

Furthermore, 2DEGs occur at LaTiO_3_/KTaO_3_ interfaces [248] as well as at epitaxial LaAlO_3_ on KTaO_3_ [249]. KTaO_3_ is a material that is very similar to SrTiO_3_ in many aspects [250,251]. Thus, KTaO_3_ also possesses promising dielectric, photoconductive and optical properties [252]. Chen et al., recently reported a 2D superconductivity (T_c_ ~ 0.9 K) at (110)-oriented KTaO_3_ interfaces after growing amorphous LaAlO_3_ films on KTaO_3_ single crystal substrates using PLD (Figure 8g) [253]. The occurrence of a 2DEG is most likely due to oxygen vacancies. Higher numbers of 2D layers, thinner superconducting layer thickness and higher density of disorders contribute to an increase in T_c_. The midpoint T_c_ of the heterostructure, defined as 50% normal-state resistance, is approximately three times larger than that in LaAlO_3_/SrTiO_3_ interfaces [9,254,255], making fundamental studies and technical applications of superconducting 2DEGs more easily accessible.

Liu et al., confirmed the results with the discovery of superconducting PLD grown interfaces between (111)-oriented KTaO_3_ and insulating overlayers of LaAlO_3_ [256]. It was found that the superconductivity of this heterostructure exhibits a crystallographic direction dependence in contrast to 2DEGs at SrTiO_3_ interfaces [9,254,255] and can be continuously tuned from superconducting into insulating states by applying a gate voltage [257]. Temperature-dependent sheet resistance R_sheet_ (T) indicates the occurrence of superconductivity with a midpoint T_c_ ~ 2 K (Figure 8h). The normal-state Hall resistance indicates electrons as charge carriers rather than holes (Figure 8i) with a carrier density much higher than that of a typical LaAlO_3_/SrTiO_3_ interface. In this case, it seems that the decisive parameter is not the number of two-dimensional layers, but the mobility of charge carriers. LaAlO_3_/KTaO_3_ interfaces thus offer ideal conditions to investigate physical phenomena of 2D superconductors.

Table 1 summarizes published material combinations of bilayers and their most important properties.

By stacking one unit cell BaTiO_3_ and one unit cell SrTiO_3_, Jia et al., succeeded in modifying the electronic properties of both starting materials with respect to a reduced band gap and a strain-dependent in-plane ferroelectric polarization [73]. Thin-film solar cells based on mixed organic-inorganic halide perovskites show a power conversion efficiency of 20% thanks to their high optical absorption coefficient and strong luminescence and are therefore of great interest for technological applications [285]. In the field of electric energy storage and supply devices, film capacitors exhibit the highest energy density thanks to extreme charging and discharging speeds [286,287,288,289,290], enabling an ultra-high efficiency of approximately 81% in 0.88(BaTiO_3_)/0.12(Bi(MgTiO_3_)) films at room temperature [102]. Zhao et al., found that epitaxial 0.5(BiFeO_3_)/0.5(Sm_2_O_3_) composite films grown by PLD exhibited a 2–3 orders of magnitude reduction in leakage current density compared to the pure BiFeO_3_ films [291]. In agreement with previous studies of BiFeO_3_/CoFe_2_O_4_ and BaTiO_3_/Sm_2_O_3_ [292,293], they concluded that vertical interfaces, defined as interfaces between two phases in vertically aligned composite films, are the dominant conduction path as they attract oxygen vacancies.

**Figure 9:**
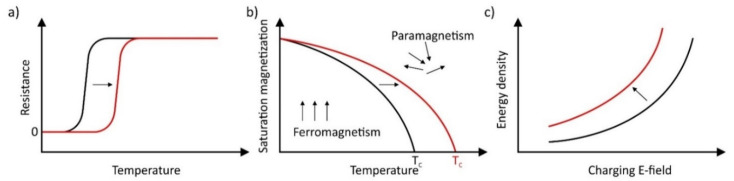
Schematic representation of the effect of combining ultra-thin layers into bilayers (red curve) on (**a**) superconductivity, (**b**) Curie temperature T_c_ and (**c**) energy density compared to bulk materials or free-standing layers (black curve).

Figure 9 schematically illustrates the influence of the arrangement of thin layers as bilayers on selected properties in comparison to bulk materials or monolayers.

An additional modification of the heterostructure properties results from the twisting of one monolayer relative to another. In contrast to conventional epitaxially grown heterostructures, the twisting angle between two free-standing membranes can be arbitrarily adjusted. Due to the rotational misalignment, the electrons in the material are trapped in periodic energy fields called moiré potentials [20]. Such superlattices affect the electronic band structure of the material and have been shown to lead to altered transport properties in bilayered graphene [50,294,295,296]. Similar phenomena occur when stacking different types of materials into atomically thin heterostructures with the incorporation of a lattice mismatch [297], when selectively incorporating defect structures [204] and when creating highly organized lamellar nanostructures and superlattices [58].

### 4.3. Multilayers

**Figure 10:**
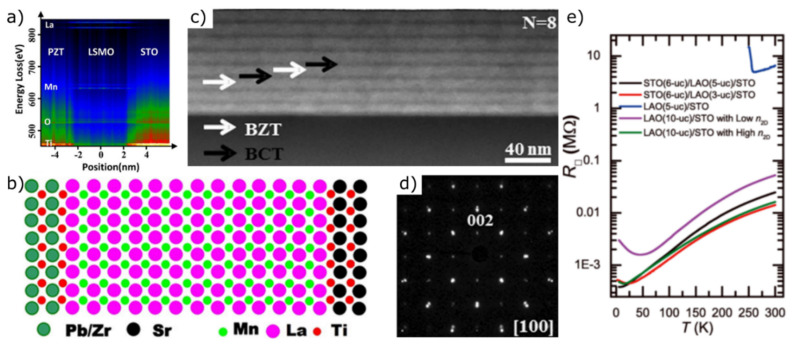
(**a**) Core-loss EELS spectrum image of Ti L, O K, Mn L and La M edge and (**b**) atomic schematic model of a Pb(Zr_0.2_Ti_0.8_)O_3_/La_0.8_Sr_0.2_MnO_3_/SrTiO_3_ structure [298]. (**c**) Cross-sectional STEM image and (**d**) SAED pattern of a (Ba_0.7_Ca_0.3_TiO_3_/BaZr_0.2_Ti_0.8_O_3_)_4_ multilayer. Black and white arrows indicate Ba_0.7_Ca_0.3_TiO_3_ (BCT) and BaZr_0.2_Ti_0.8_O_3_ (BZT), respectively [299]. (**e**) Temperature-dependent sheet resistance R_S_ for LaAlO_3_(5-uc)/SrTiO_3_(100), LaAlO_3_(10-uc)/SrTiO_3_(100), and SrTiO_3_(6-uc)/LaAlO_3_(t-uc)/SrTiO_3_(100). Samples with the SrTiO_3_ capped layer show metallic behaviour down to 5 K even with a thickness of only 3 unit cells of the LaAlO_3_ layer [242]. Figure 3a reprinted with permission from Singh et al. [242]. Copyright 2021 by the American Physical Society.

Stacking at least three layers of thin films leads to the formation of so-called multilayers, which are characterized by exotic properties due to their larger number of interfaces. In 2019, Meng et al., established a method to analyze the change in electron density at the interface of multilayer thin films. For this purpose, they prepared Pb(Zr_0.2_Ti_0.8_)O_3_/4.8 nm La_0.8_Sr_0.2_MnO_3_/SrTiO_3_ films by MBE and off-axis magnetron sputtering to investigate their electronic properties [298]. The results extracted from the core-loss EELS spectrum (Figure 10a) confirm the epitaxial growth mode and support the atomic schematic model of the heterostructure (Figure 10b).

Sandwich-structured SrTiO_3_/BiFeO_3_/SrTiO_3_ thin films show improved crystallization quality and excellent temperature stability of the dielectric constant. Their enhanced energy storage density results from the increased number of interfaces and makes the heterostructure a cost-effective and efficient option for electrostatic energy storage applications [300]. Sun et al., prepared (Ba_0.7_Ca_0.3_TiO_3_/BaZr_0.2_Ti_0.8_O_3_)_N_ multilayer structures with *n* = 2, 4, 8 by magnetron sputtering. The microstructural quality of the multilayers was investigated using STEM (Figure 10c) and SAED (Figure 10d), thereby confirming the epitaxial orientation. Apart from an increase in breakdown strength with increasing number of interfaces, an energy storage density of 52.4 J cm^−3^ has been achieved for *n* = 8 multilayers. This value is significantly higher in comparison to other Pb-free materials and comparable to many Pb-based systems [102,286,287,288,289,290,299,301,302,303,304,305,306,307,308,309].

Singh et al., investigated structural and magnetotransport properties of MBE grown SrTiO_3_-capped and uncapped LaAlO_3_/SrTiO_3_ (100) heterostructures by tuning their sheet densities and mobilities through the electrical gating effect and the layer thickness. The results reveal a conductive interface down to 5 K by using a SrTiO_3_-capped layer compared to uncapped samples with high and low sheet density (n_2D_) (Figure 10e).

## 5. Future Perspectives

The variety of property phenomena that occur when the film thickness is reduced and approaches a monolayer emphasizes the potential of atomically thin films and provides access to a wide range of applications including optoelectronics, nanoelectronics and spintronics. Thin films could be used, for example, in piezoelectric applications such as pressure sensors [310], transducers [311], high-voltage generators [312] and nonlinear energy harvesters [313], meeting demands for minimal dimensions, weight reduction and lower energy consumption. Similarly, 2D electronics like high-power transistors or ferroelectric capacitors and memory device applications are conceivable [104,314,315]. Challenges such as defect structure, processability and long-term stability of the devices must first be solved to fully exploit the potential of ultra-thin heterostructures and enable their use as transistors, semiconductor circuits or flexible and transparent electronics [240]. To solve the scalability challenge, approaches are already being pursued to increase the throughput of PLD [316,317] or to rely on MBE as an alternative method [318,319,320].

The 2D materials presented, as well as the methods for their production, also make an expansion of ultra-thin layers to other, not yet investigated materials, seem realistic for the future. The development of further monolayers in conjunction with the discovery of novel properties thus promises an even more versatile application potential. Although cuprates are those superconductors with the highest critical temperature, there is hardly any relevant literature addressing the transfer of thin film technologies to this class of materials [321,322,323,324,325] although initial investigations have shown promising results [326]. Oxide-based cuprates consist of layers of superconducting planes of copper oxide alternating with layers of other metal oxides containing ions such as lanthanum, barium and strontium. Due to the high structural similarity to the perovskite structure, an application of existing technologies to produce, characterize and apply cuprate monolayers seems promising. Physical properties such as conductivity could be targeted via chemical composition, cation substitution or oxygen content.

In addition, the use of different simulation methods offers far-reaching possibilities to predict the influence of the reduction of the layer thickness down to the monolayer limit on the resulting material properties. By using artificial intelligence and machine learning, it also seems possible to make predictions about the feasibility of producing ultra-thin layers that would extend conventional bottom-up processes. Furthermore, completely new material combinations and twists of the layers involved could be considered to predict their influence on various interfacial properties. Consequently, complex problems could be solved more efficiently by combining theoretical and experimental disciplines with numerical methods.

## Figures and Tables

**Table 1:** Property phenomena in bilayered heterostructures.

Bilayer System	Phenomena and Related Properties	Reference
Sr_2_TiO_4_/SrTiO_3_	Dynamic rearrangement during the growth of layered A_n+1_B_n_O_3n+1_ oxide systems	[258]
LaTiO_3_/SrTiO_3_	Highly active participation of a TiO_2_ adlayer in dynamic layer rearrangement; magnetotransport properties; formation of a highly mobile conduction channel	[259,260]
LaFeO_3_/SrTiO_3_	Dynamic interfacial rearrangement of atomic planes as a function of substrate termination; band alignment affected by interfacial polarity; photocurrent-voltage curves depending on interface termination; spontaneous polarization evoking photovoltaic properties	[261,262,263,264]
TiO_2_/SrTiO_3_	Formation of a defect-free zone and an amorphous boundary layer caused by differences in chemical potential and defect mobilities of both phases	[265,266]
CeO_2_/SrTiO_3_	Defect trapping by atomic interface steps leading to localized amorphization under ion radiation	[267,268]
MgO/SrTiO_3_	Orientation-specific amorphization and intercalated recrystallization at ion-irradiated interfaces	[269]
GdTiO_3_/SrTiO_3_	Magnetic order; electrical transport and Mott insulation properties, Curie temperature of 30 K; intrinsic electron reconstruction; high concentration of mobile carriers (2DEG); interfacial polar discontinuity	[270,271,272,273,274,275,276]
NdTiO_3_/SrTiO_3_	Ultra-high carrier densitiy regime due to additional charge transfer from band alignment	[277]
γ-Al_2_O_3_/SrTiO_3_	High quality epitaxial heterointerface (Figure 8j), high electron mobility; quantum magnetoresistance oscillations; band bending and alignment, thickness-dependent transport properties (Figure 8k,l)	[141,278,279]
LaCrO_3_/SrTiO_3_	Unexpected formation of 2DEG at the initially insulating interface	[280]
LaVO_3_/SrTiO_3_	Interface conduction based on electronic reconstructions; thickness-dependent metal-insulator transition; Hall effect at low temperature; growth rate and substrate temperature-dependent structural and electrical interface properties by oxygen substrate-to-film transfer	[281,282]
SmTiO_3_/SrTiO_3_	Non-Fermi liquid behaviour; very high film carrier densities	[283]
La_0.5_Zr_0.5_O_1.75_/LaAlO_3_, Nd_0.5_Zr_0.5_O_1.75_/LaAlO_3_	Dynamic self-assembly during growth creates coherent interfaces between oxide materials of different crystal structure	[284]
